# Aspiration of tracheoesophageal prosthesis in a laryngectomized patient

**DOI:** 10.1186/2049-6958-7-25

**Published:** 2012-08-09

**Authors:** Sergio C Conte, Elena De Nardi, Federico Conte, Stefano Nardini

**Affiliations:** 1 Pneumology Unit, Vittorio Veneto Hospital, Via Forlanini, Vittorio Veneto (TV), Italy; 2 Otolaryngology Head and Neck Surgery, Vittorio Veneto Hospital, Vittorio Veneto, (TV), Italy; 3 University of Parma, School of Medicine, Parma, Italy

**Keywords:** Flexible bronchoscopy, Foreign body aspiration, Tracheoesophageal prosthesis

## Abstract

**Background:**

The voice prosthesis inserted into a tracheoesophageal fistula has become the most widely used device for voice rehabilitation in patients with total laryngectomy.

**Case presentation:**

We describe a case of tracheoesophageal prosthesis’ (TEP) aspiration in a laryngectomized patient, with permanent tracheal stoma, that appeared during standard cleaning procedure, despite a programme of training for the safe management of patients with voice prosthesis.

**Conclusions:**

The definitive diagnosis and treatment were performed by flexible bronchoscopy, that may be considered the procedure of choice in these cases, also on the basis of the literature.

## Case presentation

In July 2011 a 63 year old man with a total laryngectomy was admitted to the Department of Otolaryngology Head and Neck Surgery at Vittorio Veneto Hospital for suspected aspiration of tracheoesophageal prosthesis.

The patient underwent total laryngectomy for advanced squamous cell carcinoma of the larynx eighteen months before this admission.

He was an engineer**,** smoked 60 pack/years, and usually drank half litre of wine daily.

The disease had been diagnosed two years before and the patient had been treated in the first place with radiotherapy exclusively. Seven months after the end of the treatment, in January 2010, he underwent emergency tracheostomy for acute dyspnea due to recurrence of larynx carcinoma: then he underwent total laryngectomy with primary insertion of tracheoesophageal prosthesis.

During his hospital stay he started voice rehabilitation with speech therapist for about four days.

When the patient arrived at our department in July 2011 he referred that the TEP had torn away during daily cleaning procedure, so he tried to replace the device but the attempt was unsuccessful.

The lost TEP was a classic 10 mm Blom Singer Indwelling prosthesis, changed 2 months earlier (s 1).

**Figure 1:**
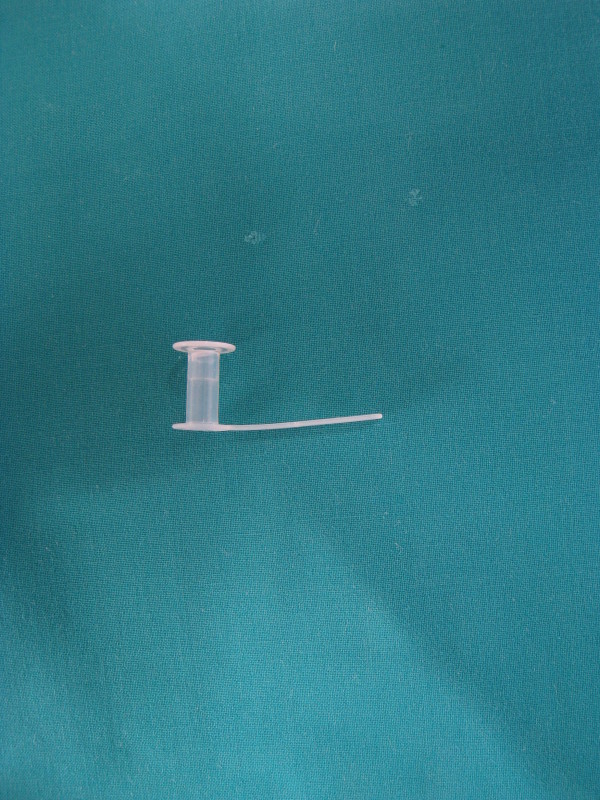
Classic 10 mm Blom Singer Indwelling voice prosthesis.

At the admission he was asymptomatic, in particular he had no dyspnea, stridor, fever or cough.

**Figure 2:**
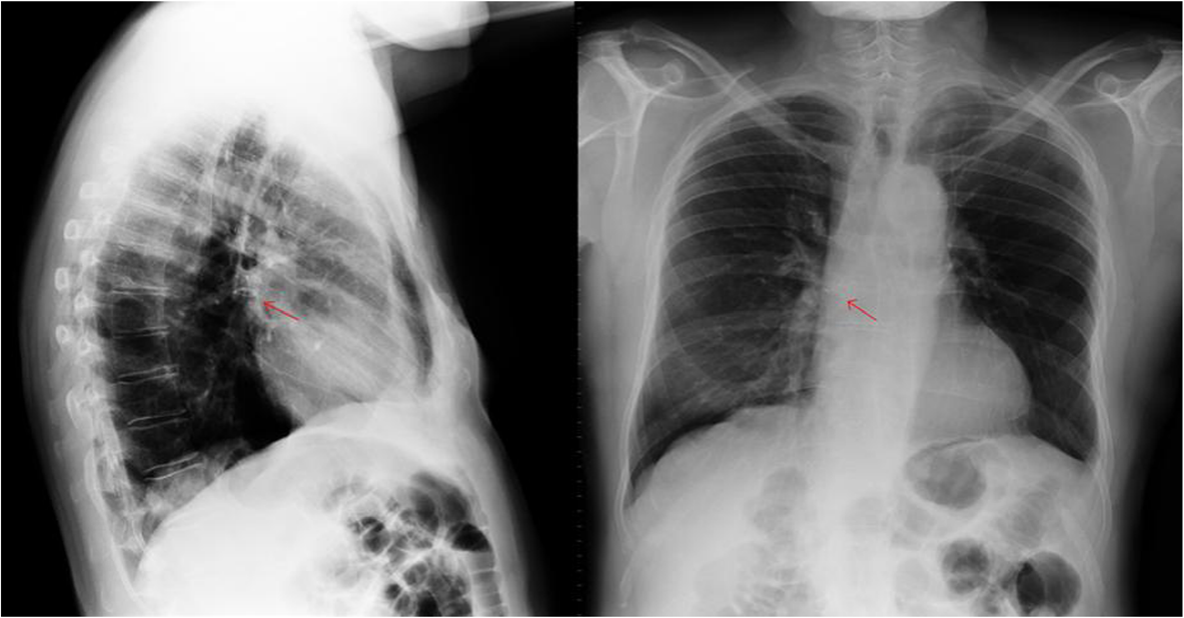
Chest radiograph showing a ring-shaped opacity in the right mainstem.

Anyway, at the examination the prosthesis was absent and he underwent immediately standard 2 view chest radiography, which demonstrated a ring-shaped opacity placed in the right lower lobe, suspicious for the aspirated TEP (Figure 2).

Thus it was planned to remove the foreign body with fiberoptic flexible bronchoscopy and to place another voice prosthesis, under general anesthesia, at the same time.

**Figure 3:**
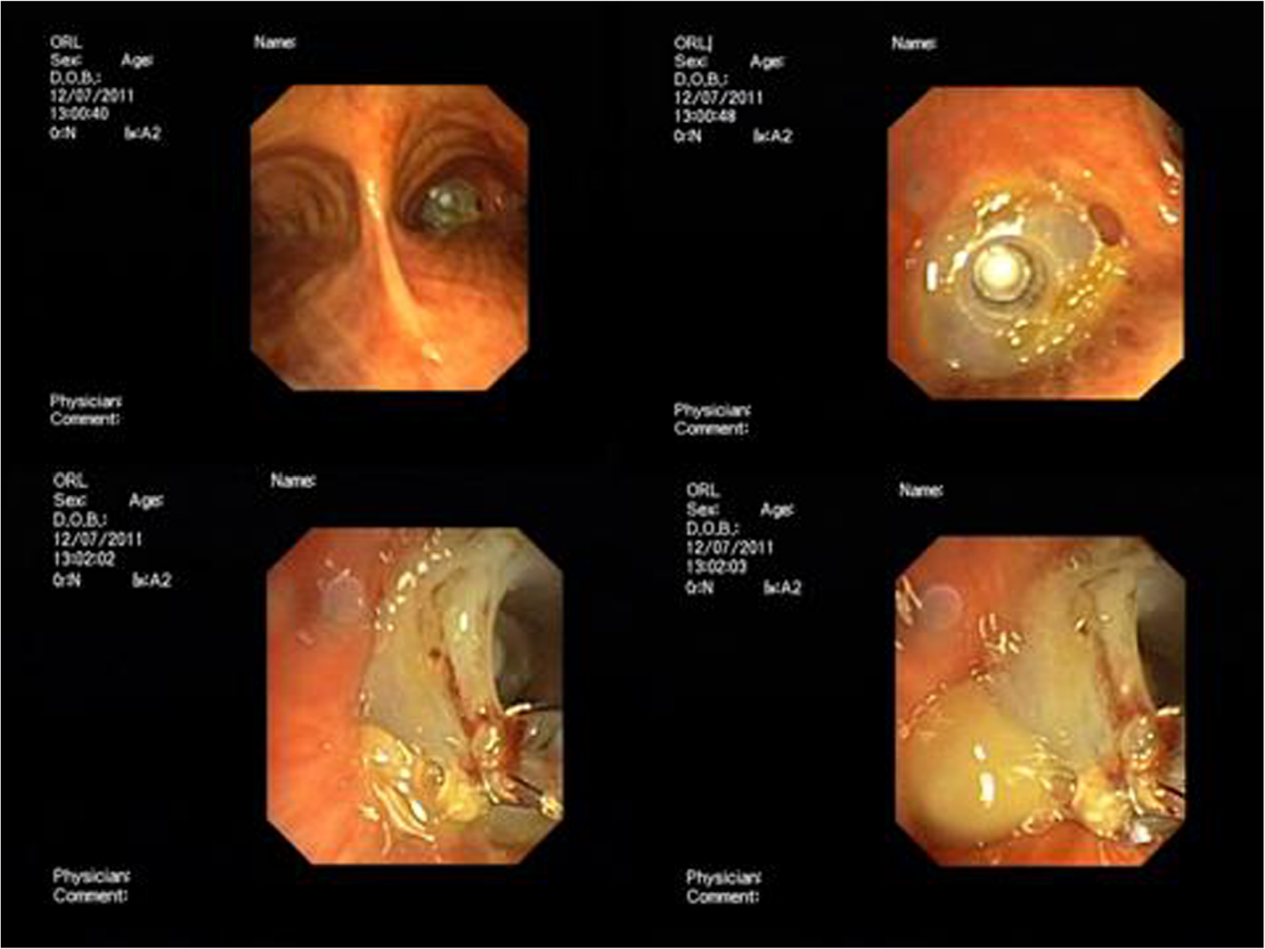
**Endobronchial foreign body.** Upper left: panoramic view from the carina. Upper right: Blom-Singer prosthesis in the bronchus intermedius. Lower left and right: TEP retrieved with biopsy forceps.

The flexible bronchoscope (Olympus BF 1 T 180) was inserted through the tracheal stoma. The TEP was present in the bronchus intermedius, just distal to its origin and it was retrieved without difficulty by grasping it with biopsy forceps (Figure 3), then withdrawing the whole bronchoscopy out of the tracheal stoma.

After removing the TEP copious issue secretions filling the bronchus intermedius were suctioned and sent for culture.

The patient was discharged the day after with prescription of antibiotic treatment.

## Discussion

Total laryngectomy provides oncologic control of hypopharyngeal and advanced laryngeal cancers that are not treatable with conservative procedures.

In these patients tracheoesophageal prosthesis (TEP) represents an integral aspect of speech rehabilitation: a fistula is surgically created between trachea and esophagus, where the voice prosthesis, a one way speaking valve, is placed.

The patient usually follows speech rehabilitation together with the speech therapist for some days and TEP is periodically changed in the ENT ambulatory (when it is broken or too long).

The authors of this technique were Singer and Blom in 1979 and it has become the most widely used device for voice rehabilitation.

This procedure has some complications that can be divided in early, that is within the first week, and late, after the first week [1].

Early complications include bleeding, edema, infection, mediastinitis, abscess, and salivary or food leakage around prosthesis.

Late complications are the formation of granuloma around the prosthesis, salivary leakage, dislocation or aspiration of the prosthesis, and fungal infection.

Permanent tracheal stoma is a risk factor for foreign body aspiration in the tracheobronchial tree in adults and several cases have been reported in literature.

The foreign bodies aspirated through the stoma in laryngectomized patients include safety pin, nails, wooden stick and speaking valve, piece of teeth [2]**,** and the gauze tape of tracheostomy tube neck (personal observations).

Prosthesis aspiration is a complication described in 3,9 % to 6,7 % of patients in published series [3,4]: it usually happens when patients try to replace the device and stimulate cough. The alcohol abuse can increase the risk of foreign body's aspiration, but other risk factors are recognized, such as impaired functional status, prior radiotherapy, neurological disease, loss of consciousness from trauma or sedatives intake [2-5]. To our knowledge only nine cases of voice prosthesis aspiration have been reported [2,5-8].

In six cases the voice prosthesis aspirated was retrieved by flexible bronchoscopy, and in two cases by rigid bronchoscopy, while in one case the removal of two TEP was performed both by the flexible and the rigid bronchoscope.

The clinical picture of TEP aspiration is extremely various: some patients present severe dyspnea and oxygen desaturation, others have few symptoms or are asymptomatic; many of these patients require hospitalization and antibiotics treatment. Chest radiograph can be normal or may demonstrate air trapping, an impacted foreign body, or atelectasis. HRCT may prove helpful for the diagnosis.

The flexible bronchoscopy is the first step in the evaluation of a patient with dislocated prosthesis, plays a determinant role in the definitive diagnosis, and is the method of choice for the management of TEP removal, while in some cases the rigid bronchoscopy is necessary.

The TEP is usually placed in the right main stem bronchus (bronchus intermedius), but it is also reported in the left main bronchus (three cases).

Our patient was asymptomatic and chest radiograph showed the TEP in the right main stem. It was placed in the bronchus intermedius and was removed without difficulty. Then, another voice prosthesis was placed under general anesthesia.

Whereas in children the rigid-tube remains the technique of choice to remove the majority of foreign bodies, in adults the flexible bronchoscopy may usually be used both for diagnosis and treatment. Therefore in adults and in children older than 12 years, the flexible bronchoscopy should be considered the first procedure for the diagnosis and removal of foreign bodies, except in cases of asphyxiating foreign bodies where rigid bronchoscopy should be considered first [9].

Our case demonstrates that in patients with total laryngectomy who aspirated TEP the flexible bronchoscopy can be the procedure of choice to remove the foreign body, whereas when the clinical condition of patients is critical, or when the TEP is placed in the mainstem left bronchus, the rigid bronchoscopy should be considered for TEP’s removal.

## Conclusions

The voice prosthesis' aspiration in total laryngectomy is a well recognizedrisk, anyway it can be reduced with careful patient's selection.

The ideal patient should be one motivated, mentally stable, with good eyesight and manual dexterity, able to take a safe care of the stoma and prosthesis, because he must correctly clean the voice prosthesis daily.

Tracheobronchial aspiration of TEP is a complication that should be considered in all laryngectomized patients with a missing TEP.

The flexible bronchoscopy is the gold standard for the diagnosis of voice prosthesis' aspiration and usually the method of choice for its safe removal.

## Consent

Written informed consent was obtained from the patient for publication of this case report and any accompanying images. A copy of the written consent is available for review by the Editor-in-Chief of this journal.

## Competing interests

The authors declare that they have no competing interests.
